# Acute kidney injury due to high-output external biliary drainage in a patient with malignant obstructive jaundice: a case report

**DOI:** 10.1186/s13256-019-2195-4

**Published:** 2019-08-13

**Authors:** Umesh Jayarajah, Oshan Basnayake, Pradeep Kumara Wijerathne, Sivasuriya Sivaganesh

**Affiliations:** 1 Professorial Surgical Unit, National Hospital of Sri Lanka, Colombo, Sri Lanka; 2 Department of Surgery, Faculty of Medicine, University of Colombo, Kynsey Road, Colombo 8, Western Province Sri Lanka

**Keywords:** High output biliary drain, Acute kidney injury, Case report

## Abstract

**Background:**

Persistent high output is a rare but potentially serious complication of percutaneous biliary drainage.

**Case presentation:**

A 68-year-old Sinhalese woman with a palliative self-expanding metal stent placed for an inoperable hilar cholangiocarcinoma presented with worsening obstructive jaundice. Ultrasonography showed intrahepatic duct dilatation with the self-expanding metal stent *in situ*. Since this was indicative of a blocked stent, percutaneous transhepatic cholangiogram-guided internal biliary stenting through the self-expanding metal stent was attempted and failed. Therefore, an external biliary drain was left in the dilated biliary system. Post procedure, she developed a high biliary output of 3–4 liters per day and went into oliguric acute kidney injury with metabolic acidosis, most probably due to inadequate fluid replacement and hypovolemia.

**Conclusion:**

Although the mechanism by which this occurs in some cases is unclear, early identification and prompt fluid resuscitation prevent acute kidney injury. The adoption of new strategies for internal drainage of long complex strictures will both prevent and ameliorate this problem.

## Background

The majority of patients with malignant biliary obstruction are unresectable at the time of presentation [1]. These non-resectable tumors are usually treated with palliative internal biliary stenting, failing which an external biliary drain (EBD) may be required [2, 3].

The volume of externally drained bile averages 700 ml per day with a range of 200–1600 ml [4]. However, high biliary outputs are rarely observed. We report the case of a patient with high output from a biliary drain following percutaneous transhepatic cholangiogram (PTC)-guided biliary drainage for malignant jaundice leading to hypovolemia and acute kidney injury (AKI). This case highlights the importance of early detection of high output from an EBD, careful monitoring, and aggressive fluid replacement, especially in an elderly patient.

## Case presentation

**Fig. 1:**
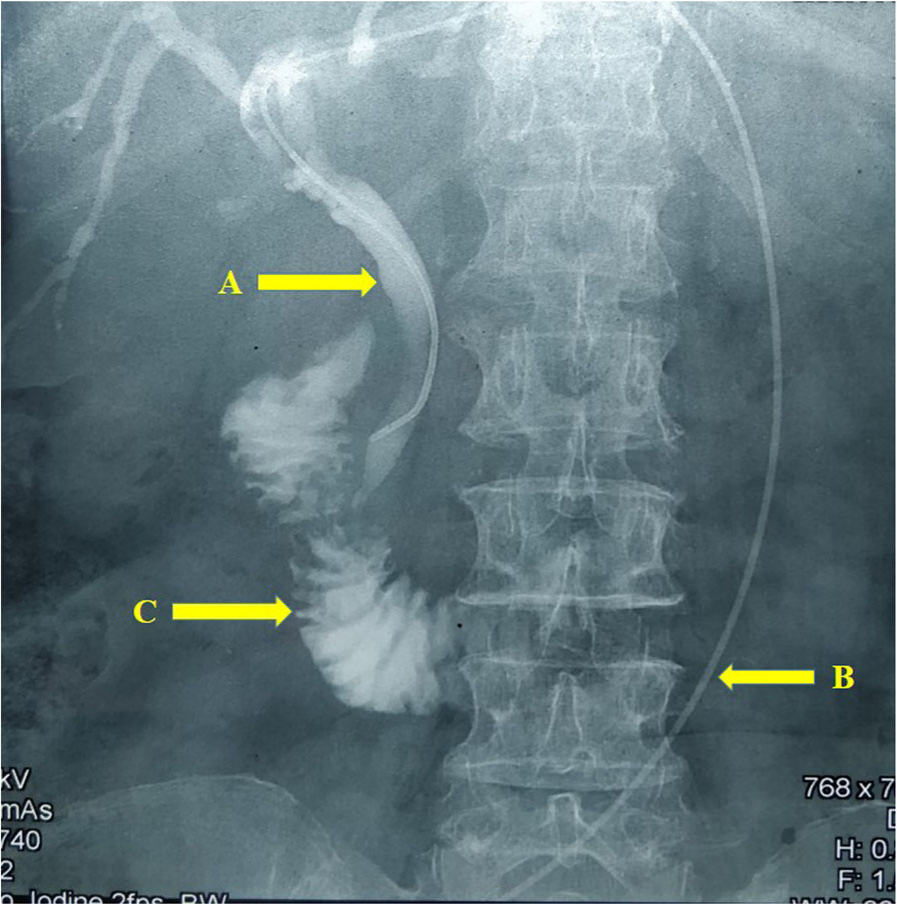
Contrast image shows a metal stent (**a**) placed through left hepatic duct, common hepatic duct, and common bile duct through guide wire (**b**). Contrast is seen in the duodenum (**c**) indicating adequate drainage

**Fig. 2:**
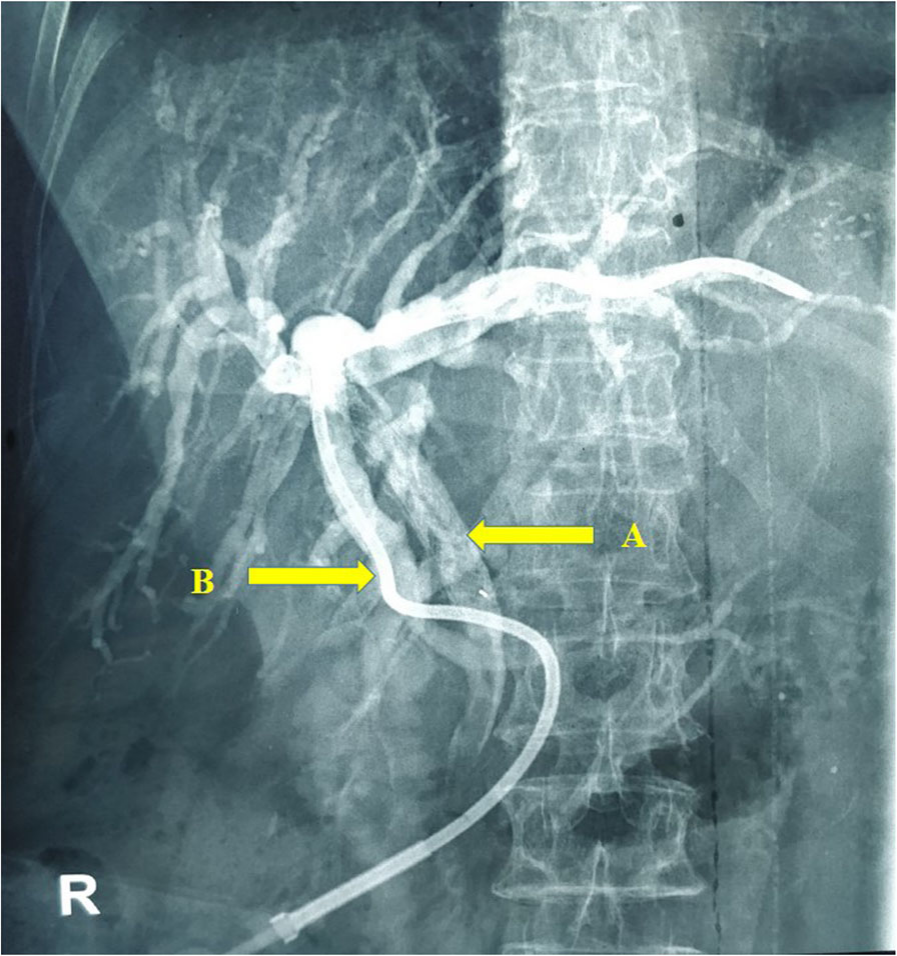
Contrast image. An external biliary drain (**b**) was inserted in the dilated left biliary duct system (8G, 25 cm). Previously inserted metal stent (**a**) is seen with some contrast entering the duodenum

A 68-year-old Sinhalese woman with a palliative self-expanding metal stent (SEMS) placed for an inoperable hilar cholangiocarcinoma a year ago (Fig. 1) presented with worsening obstructive jaundice of 2 weeks and mild cholangitis. She had poorly controlled type 2 diabetes and hypertension. She was on gliclazide 40 mg twice daily, amlodipine 5 mg twice daily, and prazosin 1 mg twice daily. She was unemployed and her social, environmental, and family history were unremarkable. She had no history of tobacco smoking or alcohol consumption. Her abdominal, respiratory, and neurological examinations were unremarkable. Her vital signs (pulse rate, 92 beats per minute; blood pressure, 130/80 mmHg; temperature, 36.8 °C), and urine output were within normal limits, but inflammatory markers were elevated (white blood cells, 11.2 × 10^9^/L; C-reactive protein, 30 mg/L). She had elevated bilirubin levels (total bilirubin, 60 μmol/L; direct bilirubin, 31 μmol/L) and low albumin levels (27.5 g/L). Her renal functions were within normal limits. Ultrasonography showed intrahepatic duct dilatation with the SEMS *in situ*. Since this was indicative of a blocked stent, PTC-guided internal biliary stenting through the SEMS was attempted and failed. Therefore, an 8G - 25 cm EBD was left in the dilated left biliary system (Fig. 2).

Post procedure, she developed a high biliary output of 3–4 liters per day and went into oliguric AKI with metabolic acidosis, most probably due to inadequate fluid replacement and hypovolemia. A cholangiogram confirmed that the EBD was in place with contrast entering the duodenum. Abdominal ultrasonography did not reveal any intra-abdominal fluid collection. The EBD was closed to control the persistently high drainage, and she underwent hemodialysis for the AKI.

Although there was temporary improvement of renal function over the next 2 weeks, she proceeded to develop gross ascites with worsening renal functions again. Decompensated cirrhosis with hepatorenal syndrome (HRS) was suspected. Her ascitic fluid was positive for coliforms suggesting superadded bacterial peritonitis. After a combination of therapy with intravenously administered antibiotics, terlipressin, and albumin she recovered and her bilirubin and creatinine levels returning to baseline. She was discharged on diuretics and antibiotic prophylaxis for spontaneous bacterial peritonitis.

On her 6-month follow-up, she was asymptomatic with marked reduction of the ascites and her renal functions were normal. Her comorbidities were well controlled.

## Discussion

We describe the occurrence of a high-output biliary drain following PTC-guided biliary drainage for malignant jaundice leading to hypovolemia and AKI. The development of decompensation of cirrhosis with HRS leading to dialysis-dependent AKI is unusual compared to previous reports.

External biliary drainage is used in circumstances where palliative endoscopic or percutaneous internal stenting for malignant obstruction has failed [2, 3]. Complications of EBD include pain, pericatheter leak, cholangitis, biliary peritonitis, hemorrhage, and, rarely, persistent high output.

This case illustrates the consequences of a lack of awareness, late recognition, underestimation of fluid depletion, and inadequate fluid replacement that led to AKI in a patient with high output from EBD. The strategy of closing the EBD to control its output probably resulted in the unforeseen yet fortuitous consequence of unblocking the pre-existing internal SEMS. It also probably contributed to the subsequent bacterial peritonitis due to a pericatheter leak of infected bile from the liver surface. The preceding series of insults probably led to hepatic decompensation with HRS and unmasked the pre-existing subclinical cirrhosis which was most likely secondary to non-alcoholic fatty liver disease (NAFLD) in the background of long-standing diabetes and hypertension.

Reports on high output from EBDs are scarce. Taber *et al*.’s review of 120 cases identified 7 who exceeded the average daily biliary output. Of them, only three exceeded outputs of more than 2 liters, as in our patient, and needed aggressive fluid therapy [5]. Similar to our case, another report also described a high output from an EBD of 3–4 liters a day complicated by pre-renal AKI which was effectively treated with octreotide and nonsteroidal anti-inflammatory drugs (NSAIDs) [6]. It is unclear from these reports or available evidence what the mechanism or risk factors for a high-output biliary drain are. However, aggressive fluid therapy, renal support with hemodialysis, octreotide, and NSAIDS were suggested to be effective in the management. The basolateral membrane of cholangiocytes contains secretin receptors which cause a cyclic adenosine monophosphate (cAMP)-dependent increase in bile release [6]. Thus, the use of octreotide was justified based on its ability to inhibit secretin-mediated bile release. Prostaglandins are essential chemical mediators in the contraction of the biliary system. Therefore, blocking prostaglandins using NSAIDS is postulated to reduce biliary contraction, potentially resulting in reduction of drain output. The presence of AKI in our patient precluded the use of NSAIDs.

This report highlights the importance of early recognition of high output from an EBD, careful monitoring, and aggressive fluid replacement to prevent AKI, especially in elderly patients. Internal biliary drainage prevents some EBD-related complications, including high output, but is a challenge in fully obstructed long segment strictures and where expertise is lacking. One promising minimally invasive strategy to prevent EBD-related morbidity and improve quality of survival in this subset of patients is the placement of endosonography-guided and fluoroscopy-guided SEMS between the left hepatic duct and the stomach, that is, a hepaticogastrostomy where the expertise and facilities are available.

## Conclusion

We described a case of persistent high output which is a rare but potentially serious complication of percutaneous biliary drainage. Although the mechanism by which this occurs in some cases is unclear, early identification and prompt fluid resuscitation prevents AKI. Aggressive fluid therapy, renal support with hemodialysis, octreotide, and NSAIDS were suggested to be effective in the management. The adoption of new strategies for internal drainage of long complex strictures will both prevent and ameliorate this problem.

## Data Availability

All data generated or analyzed during this study are included in this published article.

## Abbreviations

AKI: Acute kidney injury
cAMP: Cyclic adenosine monophosphate
EBD: External biliary drain
HRS: Hepatorenal syndrome
NAFLD: Non-alcoholic fatty liver disease
NSAIDs: Nonsteroidal anti-inflammatory drugs
PTC: Percutaneous transhepatic cholangiogram
SEMS: Self-expanding metal stent

## References

[1] Pu LZCT, Singh R, Loong CK, de Moura EGH. Malignant Biliary Obstruction: Evidence for Best Practice. Gastroenterol Res Pract. 2016;2016:3296801. 10.1155/2016/3296801 PMC4766322 26981114

[2] Knap D, Orlecka N, Judka R, Juza A, Drabek M, Honkowicz M, Kirmes T, Kadłubicki B, Sieron D, Baron J. Biliary duct obstruction treatment with aid of percutaneous transhepatic biliary drainage. Alexandria J Med. 2016;52(2):185–91.

[3] Chandrashekhara SH, Gamanagatti S, Singh A, Bhatnagar S. Current Status of Percutaneous Transhepatic Biliary Drainage in Palliation of Malignant Obstructive Jaundice: A Review. Indian J Palliat Care. 2016;22(4):378–87. 10.4103/0973-1075.191746 PMC5072228 27803558

[4] Kamiya S, Nagino M, Kanazawa H, Komatsu S, Mayumi T, Takagi K, Asahara T, Nomoto K, Tanaka R, Nimura Y. The Value of Bile Replacement During External Biliary Drainage: An Analysis of Intestinal Permeability, Integrity, and Microflora. Ann Surg. 2004;239(4):510–7. 10.1097/01.sla.0000118594.23874.89 PMC1356256 15024312

[5] Taber D, Stroehlein J, Zornoza J. Work in progress: hypotension and high-volume biliary excretion following external percutaneous transhepatic biliary drainage. Radiology. 1982;145(3):639–40. 10.1148/radiology.145.3.7146389 7146389

[6] Tiruneh F, Awan A, Musa A, Chen D. Successful Trial of Octreotide and Ketorolac for the Management of Increased Biliary Drain Output: A Case Report. Cureus. 2017;9(7):e1421. 10.7759/cureus.1421 PMC5580974 28875094

